# InternalBrace™ for intercarpal ligament reconstruction: An “All-dorsal” variant technique with capsular preservation

**DOI:** 10.1016/j.ijscr.2025.111554

**Published:** 2025-06-22

**Authors:** Jean-Baptiste De Villeneuve Bargemon, Antoine Martins, Brieuc Monin, Elise Lupon

**Affiliations:** a International Wrist Center, Bizet Clinic, Paris, France; b Hand, Wrist and Elbow Surgery, Saint Roch Private Hospital, 99 avenue Saint Roch, 83100 Toulon, France; c Department of Hand Surgery, Private Hospital La Châtaigneraie, ELSAN, Beaumont, France; d University Institute of Locomotor and Sport (IULS), Pasteur Hospital, 30 voie romaine, 06100 Nice, France; e Laboratory of Molecular PhysioMedicine (LP2M), UMR 7370, CNRS, University Côte d'Azur, Nice, France

**Keywords:** Scapholunate, Wrist, Ligamentoplasty, Internal brace, Intercarpal dorsal ligament

Dear Editor,

Scapholunate and lunotriquetral ligament injuries are frequent contributors to wrist pain [1,2]. While numerous surgical techniques and extensive literature exist on the subject, there remains no clear consensus on the best approach for managing subacute and chronic cases without joint degeneration. Among the proposed methods, the All-Dorsal technique has demonstrated encouraging outcomes in the treatment of scapholunate instability [[1], [2], [3]]. In the classical open surgical approach, authors frequently describe the need for a dorsal capsulotomy to expose the scaphoid and lunate [2]. However, according to Loisel et al., performing a capsulotomy disrupts the ligamentous attachments between the extrinsic and intrinsic ligament complexes, particularly at the dorsal pole of the lunate [4]. Furthermore, the subsequent ligament repair does not restore preoperative stability levels [4]. Additionally, capsulotomy may damage the posterior interosseous nerve fibers, which contribute proprioceptive biofeedback to scapholunate biomechanics [4]. These findings support the rationale for an arthroscopic approach, yet such techniques demand advanced surgical skills.

To mitigate the sequelae associated with capsular opening, we proposed a variation of the classical All-Dorsal technique [2] that preserves the dorsal capsule, following the SCARE guidelines [5]. We performed this technique on two cadavers (four hands). Using a 3.5 cm approach distal to Lister's tubercle, we carefully dissected down to the dorsal capsule, ensuring its complete preservation. The remainder of the procedure followed the classical description but above the dorsal capsule [2], including guide wire reduction of the scapholunate space and fixation with FiberTape® and anchors *(Arthrex®, Naples, Florida, United States)* (Fig. 1). The key modification in our approach was identifying anchor points under fluoroscopic guidance to minimize exposure and prevent capsular damage. As a result, all reconstruction hardware remained extra-articular, preserving the dorsal capsule (Fig. 2). Maintaining capsular integrity could incorporate the dorsal capsule into the repair through plication. Stability was assessed intraoperatively using mechanical constraint maneuvers under direct visualization, and in all cases, the construct demonstrated good apparent stability. We hypothesize that this more conservative surgical alternative may prevent further destabilization of an already biomechanically compromised scapholunate complex. Capsular preservation could enhance long-term durability by eliminating the additional stress induced by capsulotomy.

**Fig. 1:**
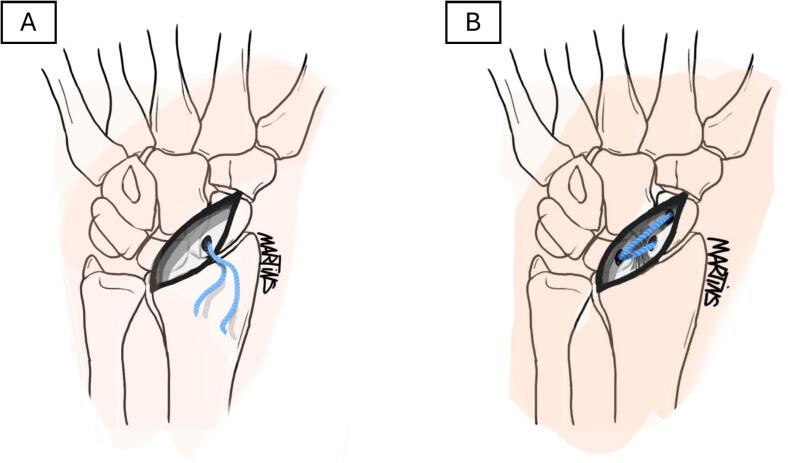
Schematic illustration of the “All-Dorsal” variant technique using an InternalBrace™ *(Arthrex®, Naples, Florida, United States)* for intercarpal ligament reconstruction. A. The first anchor securing the FiberTape*®* is inserted into a pre-drilled hole in the proximal part of the scaphoid, identified fluoroscopically, above the capsule. B. The second anchor is placed centrally in the lunate, and the third at the distal pole of the scaphoid. Both are inserted above the capsule under fluoroscopic guidance.

**Fig. 2:**
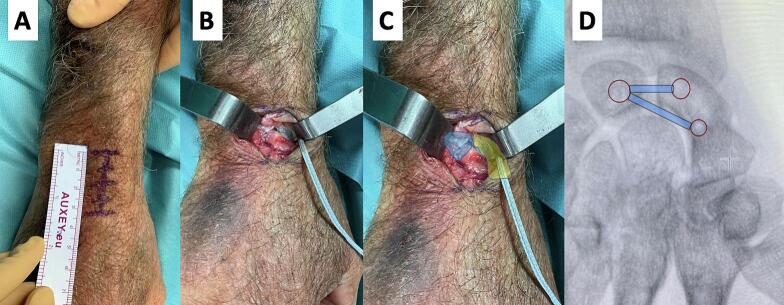
Variation of the surgical technique with preservation of the dorsal wrist capsule in a cadaveric specimen. A. 3.5 cm distal approach to Lister's tubercle B. InternalBrace™ reconstruction (“All-Dorsal”) performed according to the surgical variant. C. Overlay of the key bony structures (blue: lunate, yellow: scaphoid). D. Intraoperative fluoroscopy at the end of the procedure with a schematic overlay illustrating the placement of the InternalBrace™ on the carpal bones. (For interpretation of the references to colour in this figure legend, the reader is referred to the web version of this article.)

One potential limitation of this approach is that preserving the capsule may prevent adequate removal of fibrotic tissue, which is particularly relevant in cases of static scapholunate injuries. In such cases, arthroscopy prior to ligamentoplasty may be beneficial, both to confirm the indication for surgery (e.g., to assess osteoarthritis) and to convert static lesions into dynamic ones through aggressive debridement [6]. However, performing the entire procedure arthroscopically remains technically demanding, especially during anchor placement, potentially limiting its widespread adoption among surgeons less experienced in wrist arthroscopy [3].

We propose this modified All-Dorsal technique as a potential solution for preserving the dorsal capsule without requiring advanced arthroscopic expertise. However, our findings are based on cadaveric specimens and have not been tested in clinical practice. Further biomechanical studies under stringent loading conditions are necessary before considering clinical application.

## CRediT authorship contribution statement

**J-B. de Villeneuve Bargemon** conceived of the presented idea wrote the first draft of the manuscript, and collected data.

**A. Martins** drew Fig. 1 and reviewed the final manuscript.

**B. Monin**, **E. Lupon** helped to write the manuscript and collected data.

**E. Lupon** supervised the findings of this work.

## Consent

All patients had signed informed consent for anonymous use of their data and for body donation to science while they were alive.

## Ethical approval

This study was performed in line with the principles of the Declaration of Helsinki. This study was approved by the Nice institution's Research Ethics Committee on February 31th 2025.

## Guarantor

All authors in the article accept full responsibility for the work, have access to the patient's information, and decide to publish.

## Research registration number

1. Name of the registry: IORG0012275 - iULS-University Institute for Locomotion and Sport

Cadaver study

3.Hyperlink to your specific registration (must be publicly accessible and will be checked): N/A It is not the first in Man case report.

## SCARE guideline

The work has been reported in line with the SCARE criteria 2025.

## Funding

The authors declare that no funds, grants, or other support were received during the preparation of this manuscript.

## Declaration of competing interest

None.

The authors declare no conflicts of interest.

The authors have nothing to disclose.
